# Using Microbial Responses Viewer and a Regression Approach to Assess the Effect of pH, Activity of Water and Temperature on the Survival of *Campylobacter* spp.

**DOI:** 10.3390/foods11050637

**Published:** 2022-02-22

**Authors:** Hayrunisa Icen, Maria Rosaria Corbo, Milena Sinigaglia, Burcu Irem Omurtag Korkmaz, Antonio Bevilacqua

**Affiliations:** 1 Department of Nutrition and Dietetics, Faculty of Health Sciences, Marmara University, Maltepe, Istanbul 34854, Turkey; hayrunnisaicen@hotmail.com; 2 Department of Agriculture, Food, Natural Resources and Engineering (DAFNE), University of Foggia, Via Napoli 25, 71122 Foggia, Italy; mariarosaria.corbo@unifg.it (M.R.C.); milena.sinigaglia@unifg.it (M.S.)

**Keywords:** *Campylobacter jejuni*, Weibull modelling, predictive microbiology, regression

## Abstract

This study aimed at developing a model for evaluating the survival of various *Campylobacter jejuni* strains under different conditions in culture media and poultry data from ComBase. *Campylobacter* data of culture media (116) and poultry (19) were collected from Microbial Responses Viewer, an additional tool of ComBase. The Weibull equation was selected as a suitable model for the analysis of survival data because of the nonlinearity of survival curves. Then, the fitting parameters (first reduction time and shape parameter) were analysed through a Kruskall–Wallis test and box-whisker plots, thus pointing out the existence of two classes of temperature (0–12 °C and 15–25 °C) and pH (4–6.5 and 7–7.5) acting on the viability of *C. jejuni*. Finally, a general regression model was used to build a comprehensive function; all factors were significant, but temperature was the most significant variable, followed by pH and water activity. In addition, desirability and prediction profiles highlighted a negative correlation of the first reduction time with temperature and a positive correlation with pH and water activity.

## 1. Introduction

*Campylobacter* spp. are commensal organisms in bovine, sheep, pigs and poultry alongside various birds and usually do not cause any symptoms in animals. They are Gram-negative, slender, spirally curved rod, non-spore-forming and microaerophilic bacteria [1,2]. It is known that there are 51 species and 16 subspecies belonging to the *Campylobacter* genus of the Campylobacteraceae family. Two subspecies belong to the species *Campylobacter jejuni*: *Campylobacter jejuni* subsp. *doylei* and *Campylobacter jejuni* subsp. *jejuni* [3].

Campylobacteriosis is an infectious disease generally caused by *C. jejuni,* but *C. coli, C. concisus, C. upsaliensis, C. ureolyticus, C. hyointestinalis* and *C. sputorum* also give rise to this infection [4]. Given foodborne diseases, it is seen that *C. jejuni* and *C. coli* are the most important and most resistant to physical conditions. The most common clinical manifestations in humans consist of diarrhoea, fever, abdominal pain, headache, nausea and vomiting. However, there are complications such as Guillain–Barré syndrome after infection [5].

*Campylobacter* spp. can contaminate foods in different ways. It is known that the major contamination sources of *C. jejuni* infection in humans are poultry products [6,7]. The important step causing an increase in the thermophilic *Campylobacter* load, which is one of the clinically crucial etiologies of gastroenteritis in humans all over the world, is the transportation and slaughter of animals with intestinal carriers [2,8].

Predictive microbiology in foods is an area of applied research in food microbiology using mathematical models to predict microbial growth and responses in different environmental conditions [9,10]. Predictive models can provide accurate predictions on microbial growth and inactivation. Using these predictions instead of microbiological experiments offers a cheaper and more efficient alternative for researchers [11,12]. There are different levels of modelling in Predictive Microbiology; the classic definition is as follows:

1. Primary models, which show cell number as a function of time to model growth, inactivation or survival;
2. Secondary models (for example gamma approach, square root and polynomial equations), which focus on some parameters of primary models (e.g., growth or inactivation rate, lag phase) as a function of intrinsic or extrinsic factors (pH, activity of water, temperature, salt, the concentration of antimicrobial compounds, etc.)
3. Tertiary models, which are databases and software able to simulate a priori growth or inactivation as a function of some input conditions.

The most known database or tertiary model is ComBase, which includes more than 60,000 records on microorganisms’ behaviours in different environments and some models to predict growth and inactivation [13,14]; moreover, it also contains some additive tools, improving its performances in some fields. One of these additive tools is Microbial Responses Viewer (MRV), which is a database consisting of microbial growth/no growth data under specified environmental conditions of temperature, pH and activity of water (a_w_) derived from ComBase [15].

Although *Campylobacter* spp. are challenges in food safety, there are still a few research works that predict its survival; moreover, the research available in the literature are based on single strains or a mix composed of few isolates. Some evidence is available for chicken meat during a model gastric digestion [16] to predict infections through a dose–response approach [17], inactivation during heat treatment through a Baynesian approach [18], the compliance to performance criteria in poultry meat [19] or the estimation of incidence of campylobacteriosis through a Monte Carlo simulation; however, to the best of our knowledge there are not models able to predict the effect of some intrinsic or extrinsic factors of foods on a wide range of isolates of *Campylobacter* spp. Therefore, the main aim of this research was to develop a polynomial model able to predict *C. jejuni* survival in lab media and foods (poultry), taking into account strain variability as well as evaluating the goodness and the usefulness of this model. This aim was addressed through some intermediate milestones:

(a) Using MRV to generate the data of *C. jejuni* (cell counts vs. time);
(b) Primary modelling;
(c) Building a polynomial equation through the multiple regression approach.

## 2. Materials and Methods

### 2.1. Research Planning

**Figure 1:**
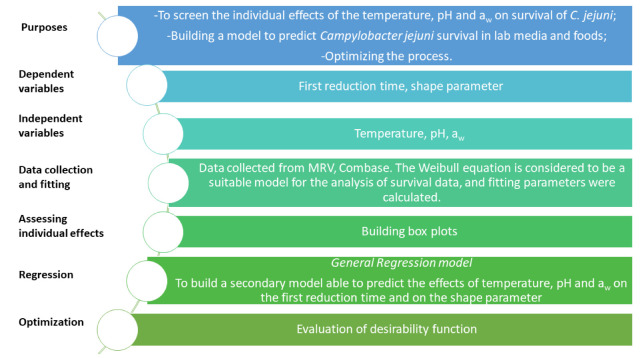
Study design.

The research focused on the building of a comprehensive model to assess the effect of pH, temperature and a_w_ on *Campylobacter* spp. The source of data was MRV, while the steps for model building are in Figure 1.

### 2.2. Data Collecting from MRV

The source of data was Microbial Responses Viewer (MRV), an additional tool of ComBase. By selecting *Campylobacter* in MRV and then a culture medium or poultry, from the general plots it was possible to gain access to the different datasets available in the model (Supplementary Figure S1/File S1). For some of them, there was only a linear death kinetic without experimental values (Supplementary Figure S2A/File S1). These combinations were excluded, while only the combinations with a scatter plot were retained (Supplementary Figure S2B). The values were pasted and copied into an Excel sheet for the second step (Supplementary File S2).

Data from both wild and collection isolates (see Supplementary Tables S1 and S2/File S1) were collected; Table 1 shows the conditions for which data were gained.

### 2.3. Primary Modelling with Weibull Function

The Weibull equation is considered to be a suitable model for the analysis of survival data, as it explains the nonlinearity often observed in survival curves [20]. This function has two parameters: shape parameter (*p*) and first reduction time (δ). The shape parameter informs about the shape of the survival curves of microorganisms and takes into account at least three shapes of the death curve: downward (*p* > 1), upward (*p* < 1) and linear (*p* = 1). The first reduction time is the time to attain a reduction of 1 log CFU/mL in the cell counts [21,22]. The δ value is similar with the D (decimal reduction time) value, but it differs from D in that the δ parameter gives information about the mean of the distribution describing the time of death of the microbial population [20].

The Weibull equation was used in the form cast by Mafart et al. [23]:

(1) $$\log N=\log N_{0}-{\left(t/\delta \right)}^{p}$$

The total data in culture media (116 datasets) and poultry (19 datasets) were fitted through Statistica software version 7.0 (Statsoft, Tulsa, OK, USA). The goodness of fitting was evaluated according to the coefficients of regression, the sum of squares error/residual sum of squares/final loss.

### 2.4. Box-Plots

The first reduction time and the shape parameter were also analyzed through the Kruskal–Wallis test (*p* < 0.05) and box–whisker plots as a function of pH, temperature and a_w_ to gain a comprehensive overview of the effects of these variables on strain survival.

### 2.5. General Regression Model

A general regression model was used to build a secondary model able to predict the effects of temperature, pH and a_w_ on the fitting parameters of Weibull function. The significance of the models and parameters was evaluated by the Sum of Squares, the Mean Sum of Squares, the R-value for multiple regression and using Fisher’s test.

The effect of each independent variable (temperature, pH, a_w_) on the fitting parameters of the death kinetic of Weibull (*p* and δ) was evaluated through the individual desirability functions, estimated as follows:

(2) $$d=\{\begin{array}{cc} 0 & y\leq y_{\min}\\ \left(y-y_{\min}\right)/\left(y_{\max}-y_{\min}\right) & y_{\min}\leq y\leq y_{\max}\\ 1 & y\geq y_{\max} \end{array}$$

where y_min_ and y_max_ are the minimum and maximum values of the dependent variable, respectively.

## 3. Results

### 3.1. Primary Model

The main assumption of the models described in this section and in the following ones is that *C. jejuni* experiences only a death kinetic, as also reported by MRV. Growth was not considered.

*Campylobacter* survival in MRV is described by a linear model; however, for several situations, the time-dependent survival kinetics of the strains cannot be explained by the linear model because there were some deviations from linearity (data not shown).

**Figure 2:**
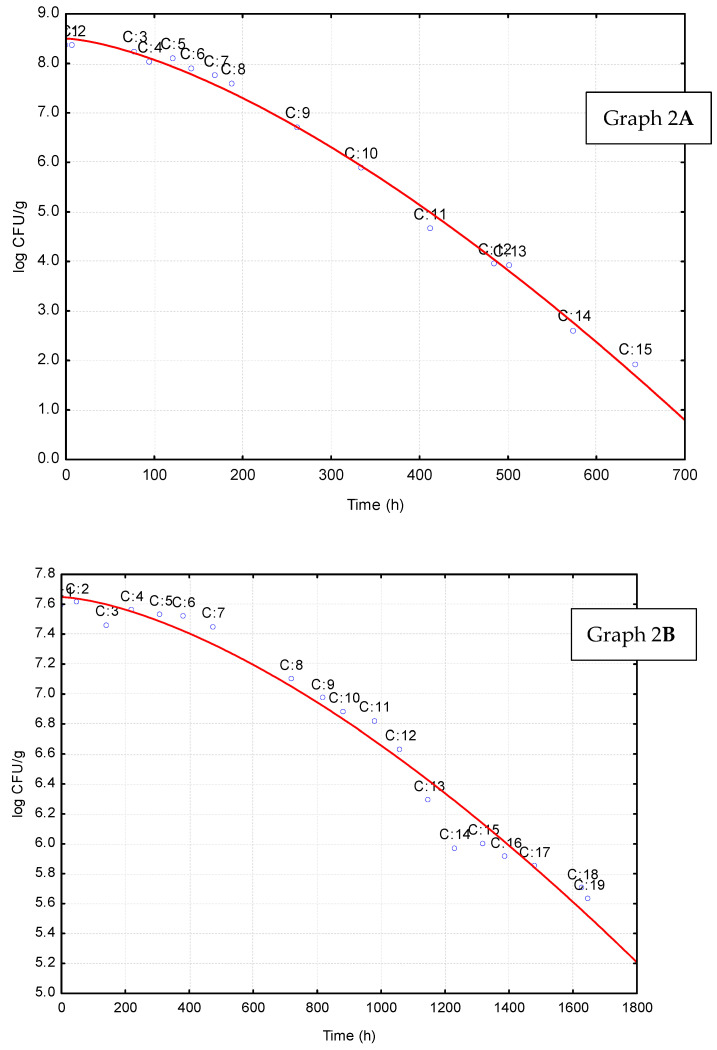
Death kinetics of *Campylobacter* spp. in different conditions (For graph 2(**A**); T = 20 °C, pH = 6.7, a_w_ = 0.997 and for graph 2(**B**); T = 4 °C, pH = 6.8, a_w_ = 0.997). Data were collected from Microbial Response Viewer, while the line represents the best fit through Weibull equation.

After a preliminary selection, the Weibull function was chosen because it is suitable to describe concave or convex decay curves of microorganisms. Most datasets from MRV, in fact, showed a concave shape for a possible shoulder effect. Biologically, it is known that the shoulder step refers to the period when microorganisms do not die yet due to various reasons [13]. As an example, Figure 2 shows two death kinetics of *Campylobacter* spp. in lab media, while in Supplementary Table S1 there are Weibull parameters and R-values for all datasets.

In culture media, the δ parameter varied as a function of temperature level, but for each temperature, a strong variability was found; mainly at 0, 4, 8 and 12 °C, the difference between the min –max values of δ were the highest. For example, it has been observed that the δ parameters for 0 °C were 37.4–955.4 h (min–max), and the min–max values for *p* parameters were 0.64 and 8.30. However, at 25˚C, it is shown that the δ parameters of the strains were between 3.22–140.79, and the *p* parameters were between 0.71–2.20.

The lowest correlation coefficient values of the Weibull equation in the culture media were 0.12416 and 0.61198, while the rest were above 0.85 (Supplementary Table S1), thus suggesting that the Weibull model could satisfactorily describe the death kinetics of this microorganism.

In poultry, while the min δ was 4.09 h, the max was 401.65 h, and the *p* parameters were in the range of 0.68–2.13. Therefore, it was observed that survival kinetics exhibited upward and downward curves similar to the culture media. It has been shown in Poultry’s data that the correlation coefficients of the Weibull equation were 0.92 and above (Supplementary Table S2).

### 3.2. Effects of pH, Temperature and a_w_

**Figure 3:**
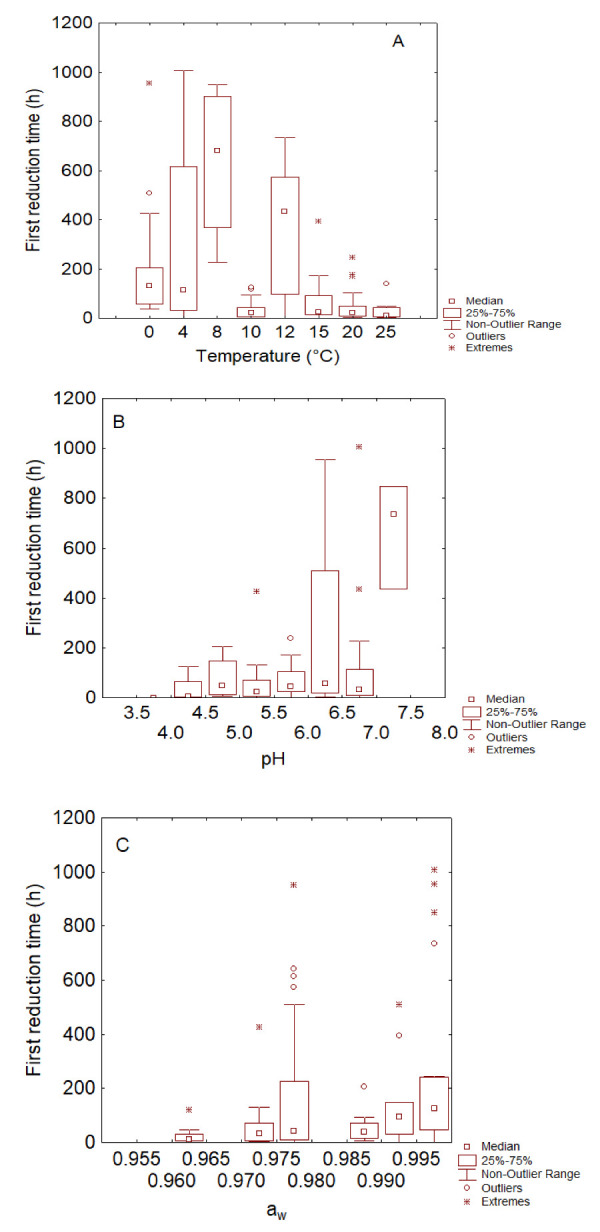
Box–whisker plots for the effects of temperature (**A**), pH (**B**) and a_w_ (**C**) on the first reduction time of *Campylobacter*.

As a first step, box–whisker plots for the effects of pH, temperature and a_w_ on the first reduction time and shape parameter were built. Temperature profiles for the first reduction time highlighted two groups (Figure 3A): the first one comprised the death kinetic at 0, 4, 8 and 12 °C with higher values of δ, although at 10 °C a statistical artifact was found due probably to a lower number of cases and datasets available in MRV. This first group was characterized by a δ-value up to 1000 h. The second group for the temperature profile (*p* < 0.05) was composed of the datasets at 15, 20 and 25 °C with a lower δ-value (<200 h).

The box plot also suggests a strong variability for each temperature due to at least two different reasons: the experiments were conducted at different conditions of pH and a_w_ and with different strains.

The pH profile of δ points out a possible effect of pH with the same limitations reported for the temperature (variability) (Figure 3B). The Kruskal–Wallis test pointed out two groups: the first one was composed of the death kinetics up to pH 6, which showed a first reduction time < 200 h, and the second one was at pH 7.0–7.5 (median value of δ at 780 h). Moreover, an intermediate group with a trend similar to pH 3.5–6.0 and 7.0–7.5 was found at pH 6.0–6.5: this transition group had a median value of δ of 80 h, similar to the group at pH 3.5–6.0, but the third quartile (500 h) and the maximum of the distribution (1000 h) suggests the existence of some strains with a trend similar to the second group (pH 7.0–7.5).

**Figure 4:**
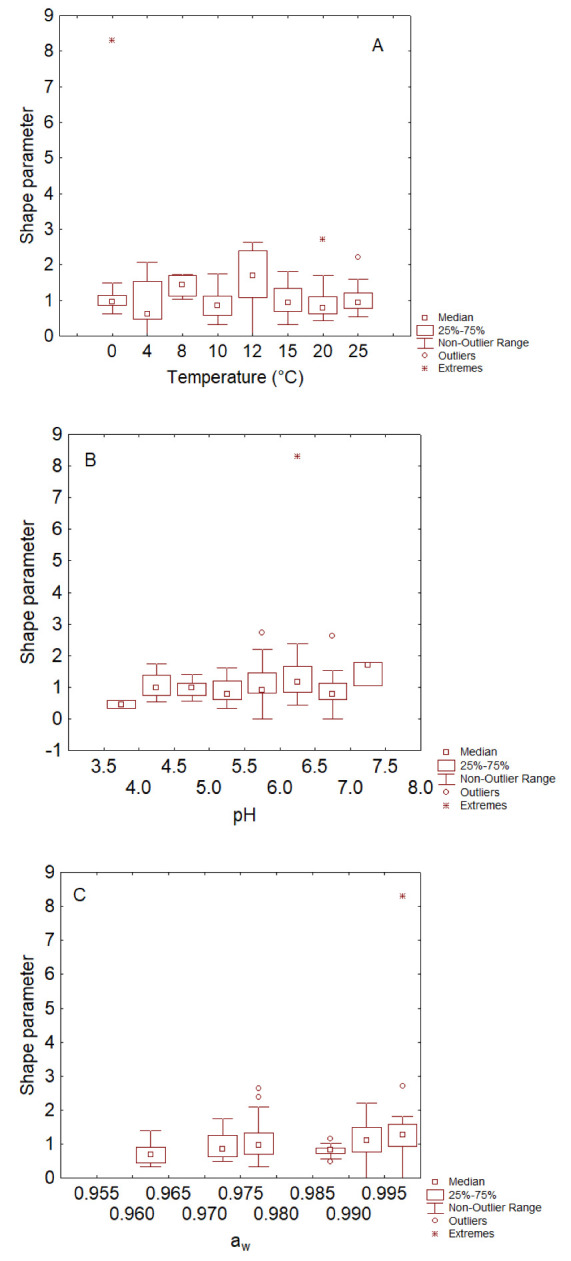
Box–whisker plots for the effects of temperature (**A**), pH (**B**) and a_w_ (**C**) on the shape parameter of *Campylobacter*.

The effect of a_w_ was less pronounced and less significant (Figure 3C), similar to the effect of the factors on the shape parameter (Figure 4). For this second parameter, a significant effect was recorded only for pH because in the range 3.5–4.0, the shape parameter was always <1, thus suggesting an upward death kinetic and the lack of a shoulder phase (or resistance period) (Figure 4B).

### 3.3. Secondary Models

In the second step of this research, a regression approach was used to assess the statistical weight of each factor (temperature, pH and a_w_) on the first reduction time and on the shape parameter; the methodology used was the general regression model.

For the first reduction time, the model highlighted the significance of all factors (pH, temperature and a_w_), although the existence of several outliers and the strong variability in some combinations pointed out only a partial correlation and a qualitative trend, rather than a quantitative function.

**Figure 5:**
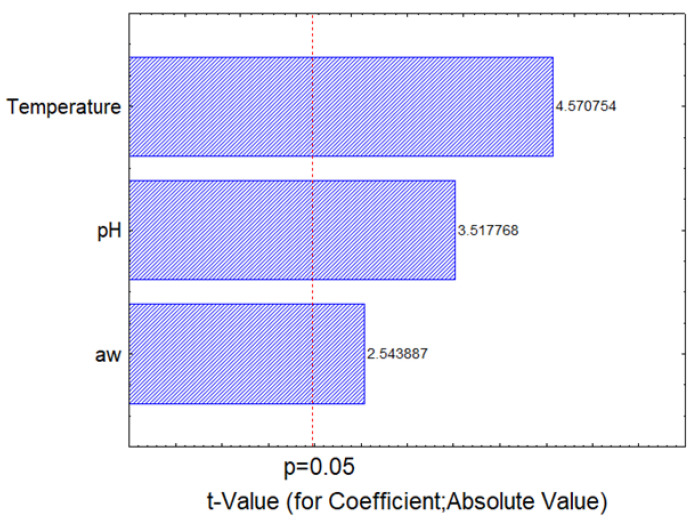
Pareto chart for the standardized effects of temperature, pH and a_w_ on the first reduction time of *C. jejuni*. The degrees of freedom of the model were 109.

Figure 5 shows the Pareto chart of standardized effect (bars); a longer bar denotes a more significant effect. Thus, the most significant term was temperature, followed by pH and a_w_. In addition, the mathematical term of the temperature was negative, while pH and a_w_ had a positive term; that is, the model predicts a decrease of the first reduction time when temperature increases, while an increase of pH and a_w_ determines an increase of this parameter.

The quantitative correlation of the shape parameter with the factors could be better highlighted by the desirability profiles. Desirability is a dimensionless parameter, ranging from 0 to 1 and is the answer to following question: how much desired is an output? The reply is 0 for the worst result (or the minimum value) and 1 for the best one (or the maximum value). Moreover, a desirability profile is often completed by a prediction profile, which shows the predicted values of the dependent variable as a function of the coded values of the factors of the design.

**Figure 6:**
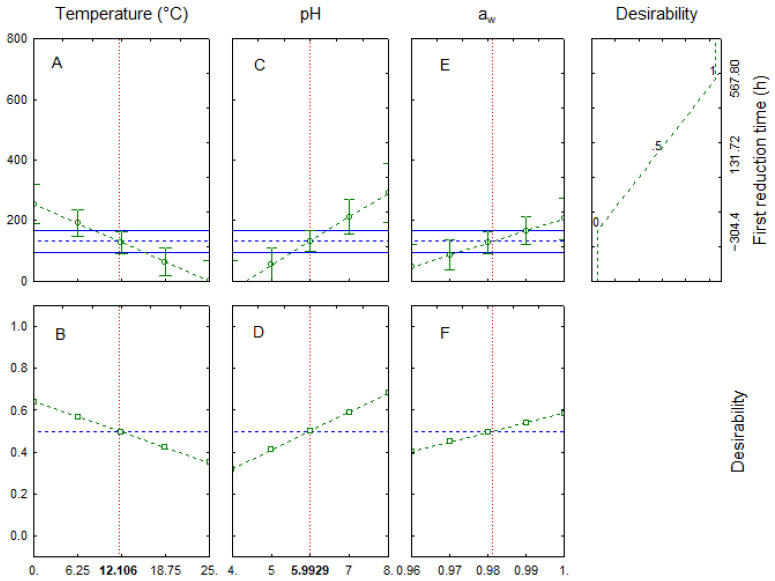
Prediction (**A**,**C**,**E**) and desirability profiles (**B**,**D**,**F**) for the effects of temperature, pH and activity water on the first reduction time of *C. jejuni*.

Figure 6 shows the desirability (A, C and E) and the prediction profiles (B, D and F) for the effects of the factors on the first reduction time. The model predicted a negative correlation of the temperature with a decrease of δ from 255 h at 0 °C (desirability at 0.64) to 0.81 h at 25 °C (desirability at 0.35) (Figure 6A,B), thus stressing the strong survival of *C. jejuni* under refrigeration.

As reported for the Pareto chart, the correlation of δ with pH and a_w_ was positive, as it increased from 0 at pH 4 to 290 h at pH 8 (Figure 6C,D) and from 47 (a_w_ 0.96) to 207 h (a_w_, 0.99) (Figure 6E,F).

The general regression approach was also used to model the shape parameter; however, this parameter was less affected by the factors of the design (data not shown).

## 4. Discussion

A model for predictive purposes could be a useful tool to increase safety and to prevent foodborne illnesses; however, to the best of our knowledge, few attempts have been made for *Campylobacter* spp. mainly to model thermal inactivation [24,25] or for a qualitative risk assessment for *Campylobacter* prevalence and diffusion in the food chain [19,26,27,28].

The first steps for a robust risk assessment are hazard characterization and exposure assessment, which rely on the definition on the growth/inactivation rate of the pathogens and on the role of intrinsic and extrinsic factors. This research was aimed at contributing to this step, focusing on both the exact definition of *C. jejuni* kinetic and the evaluation of the statistical weight of three main parameters for food preservation (pH, a_w_ and temperature).

The first question was on the shape of the survival kinetic. MRV uses the log-linear model, but the pathogen experienced a different kinetic, and the Weibull model was generally able to describe it, as also reported by González et al. [29]. A non-linear kinetic is a challenge for food preservation because it could be associated to two different phenomena: a shoulder length and a tail phase.

The shoulder is the initial phase of the death kinetic and denotes a period when a pathogen does not decrease; in the Weibull model, it is associated to a *p* > 1 and a high first reduction time, as found in most datasets. The tail (associated to *p* < 1) is also a challenge because the pathogen could experience a strong reduction in the first phases of the death kinetic and then a prolonged survival, with a residual sub-population [30]. The shape parameter and the first reduction values of *C. jejuni* found after primary modelling suggests the existence of both scenarios, depending on the strain and on the combination of pH/temperature/a_w_.

The second step to build a predictive function is secondary modelling, performed in this study through a multiple regression approach.

Some studies have shown that *Campylobacter* has a high survival capacity at low temperatures [31]. In a culture medium study, conducted with Müller Hinton agar including 2% horse blood (at +2 °C), *Campylobacter* strains were viable for at least one month under atmospheric conditions [32]. The data of this research confirmed the high viability of *C. jejuni*; the δ parameter was observed at nearly 41.9 days (1006.1 h) at +4 °C and 5.9 days (140.8 h) at 25 °C. In poultry meat, the maximum value was 11.6 days (278.9 h) at +4 °C.

Concerning the *p*-parameter (shape parameter), in a study examining the survival of different *C. jejuni* strains in high- and low-mineral drinking water at +4 °C using the Weibull model, *p* parameters ranged between 1.80 ± 0.20 and 3.00 ± 0.39 [33]. We reported in our study at +4 °C that *p* parameters were in the range of 0.00–2.08. In addition, in poultry at +4 °C, *p* parameters ranged between 0.94 and 2.13.

Besides the high survival capacity in low temperatures, the food matrices are another important parameter for survival. In a study, the influence of retail liver and meat juices on the survival of *Campylobacter* strains at +4 °C was investigated for five weeks. Strains showed higher survival in beef liver juice and chicken liver juice than beef juice, chicken juice and Müller Hinton broth [31]. Particularly, a cryoprotective effect of the liver composition is mentioned, which promotes survival at low temperatures. In terms of cold tolerance, different responses between *Campylobacter* strains were observed [34].

*Campylobacter* is known to be sensitive to acid stress, as well as to drying and low a_w_ [35]. In our study, the δ value is affected by pH and to a lesser extent by a_w_. The increase of pH extends the time of death of the bacterial cell in poultry meat. In the study of Askoura et al. [36], it was observed that the acid resistance of the microorganism increased with the change of the cell membrane composition in the presence of iron. It is known that *Campylobacter* species transform their shape from a motile spiral form to coccoid under adverse conditions and become viable but non-culturable [37].

Apart from temperature, pH and a_w_, there are other factors influencing *Campylobacter* survival, such biofilm formation and oxygen. These variables were not considered in this study because they were not described in MRV; however, they should be added in the future to a comprehensive model for *Campylobacter* along with other variables such strain difference, nutrient or antimicrobial content and structure of food matrix.

The last issue raised by this research was the strong strain variability, which should be carefully considered when building a comprehensive model to avoid fail-dangerous scenarios.

## 5. Conclusions

This study aimed to develop a predictive model on the factors affecting the survival conditions of *C. jejuni*, i.e., culture media, food type (poultry), strain variability. The Weibull model was successfully used to model *C. jejuni* survival in culture medium and poultry because the pathogen had generally a non-linear death kinetic varying from a downward to an upward shape.

In addition, the not-parametric test and the box–whisker plots pointed out the existence of two classes of the first reduction time for both the temperature and pH, that is, 0–12 °C vs. 15–25 °C and 4–6.5 vs. 7–7.5, which could qualitatively describe a longer or a shorter survival of the pathogen.

Finally, the general regression model pointed out the quantitative correlation of the first reduction time with temperature, pH and a_w_ as a prodromal step to build a comprehensive model.

Several variables affected modelling and function building:

(a) The strong variability amongst the different datasets due to the different strains and experimental conditions.
(b) The lack of a unifying design (for example a Design of Experiments) to describe the interactive factors. Model building was based on several randomized combinations available on MRV, but the lack of a geometrical or factorial scheme in the combinations did not allow an estimation of interactive effects (additive or synergistic variables). It is reasonable to imagine that the factors assessed in this research acted synergistically; thus, for a comprehensive model building, the preliminary details found in this research should be validated through a DoE able to focus also on interactive factors because mutual dependence of factors is probably more important than their liner effects.
(c) The prediction of *Campylobacter* is a challenge because it is a pathogen very difficult to study (slow growth in lab, fastidious requirements for growth, etc.). In addition, MRV does not consider several variables (among others, food structure, food components, effects of natural microbiota, oxygen and carbon dioxide in the headspace), which could strongly and significantly affect the growth/survival of this pathogen.

These issues should be taken into account to design and to develop a robust model for *C. jejuni* with practical implications; moreover, other variables should be added to the model. Nevertheless, this research could be the background for future studies because it highlighted some crucial factors to consider (kind of death kinetic, strain dependence and the role of the three main factors).

## Figures and Tables

**Table 1:** Conditions of *Campylobacter jejuni* data from Microbial Responses Viewer.

Conditions	Number of Different Levels	Range
Temperature (°C)	10 (0, 4, 5, 8, 10, 12, 15, 20, 25 and 30 °C)	0–30
pH	34 (4.1, 4.3, 4.4, 4.5, 4.6, 4.7, 4.8, 4.9, 5.0, 5.1, 5.2, 5.3, 5.4, 5.5, 5.6, 5.7, 5.8, 5.9, 6.0, 6.1, 6.2, 6.3, 6.4, 6.5, 6.6, 6.7, 6.8, 6.9, 7.0, 7.1, 7.2, 7.3 and 7.4)	4.0–7.4
a_w_	10 (0.961, 0.974, 0.977, 0.980, 0.983, 0.986, 0.989, 0.992, 0.995 and 0.997)	0.961–0.997
Poultry		
Temperature (°C)	4 (4, 5, 21 and 23 °C)	4–23
pH	3 (6.0, 6.1 and 6.5)	6.0–6.5

## Data Availability

Not applicable.

## Supplementary Materials

The following supporting information can be downloaded at https://www.mdpi.com/article/10.3390/foods11050637/s1: File S1: Supplementary Figure S1: A screenshot example of *Campylobacter* data from Microbial Responses Viewer; Supplementary Figure S2: Details from Microbial Responses Viewer’s time-dependent survival graphs (A and B) of *Campylobacter* spp.; Table S1: Wild and collection isolates of *Campylobacter jejuni* strains and their death kinetics, Weibull parameters and R-values in culture media; Table S2: Wild and collection isolates of *Campylobacter jejuni* strains and their death kinetics, Weibull parameters and R-values in poultry and File S2: Viable found in MRV (Excel Sheet).
