# Supplementary material for: Using Microbial Responses Viewer and a Regression Approach to Assess the Effect of pH, Activity of Water and Temperature on the Survival of Campylobacter spp
Source: Foods. 2022 Feb 22;11(5):637. doi: 10.3390/foods11050637 (PMC8909359; doi:10.3390/foods11050637)
Supplement: Supplementary file 1 [file foods-11-00637-s001.zip › foods-1580249-supplementary.pdf]

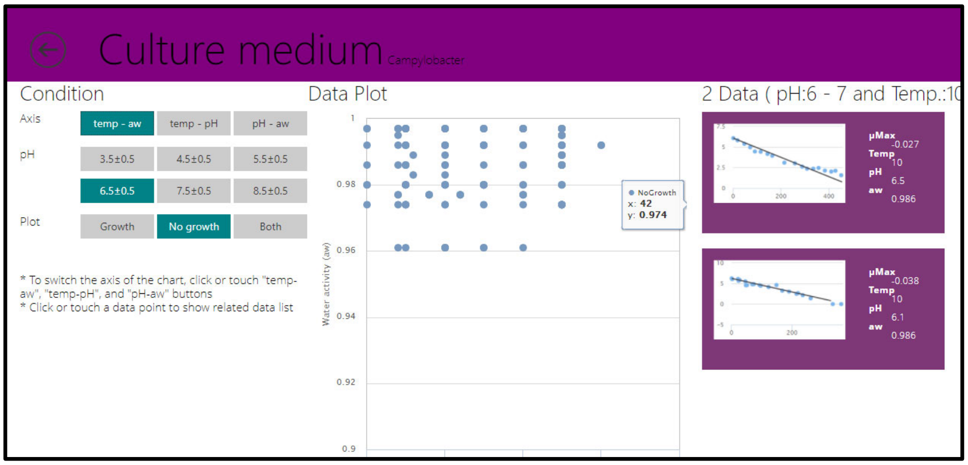

Figure S1. A screenshot example of *Campylobacter* data from Microbial Responses Viewer.

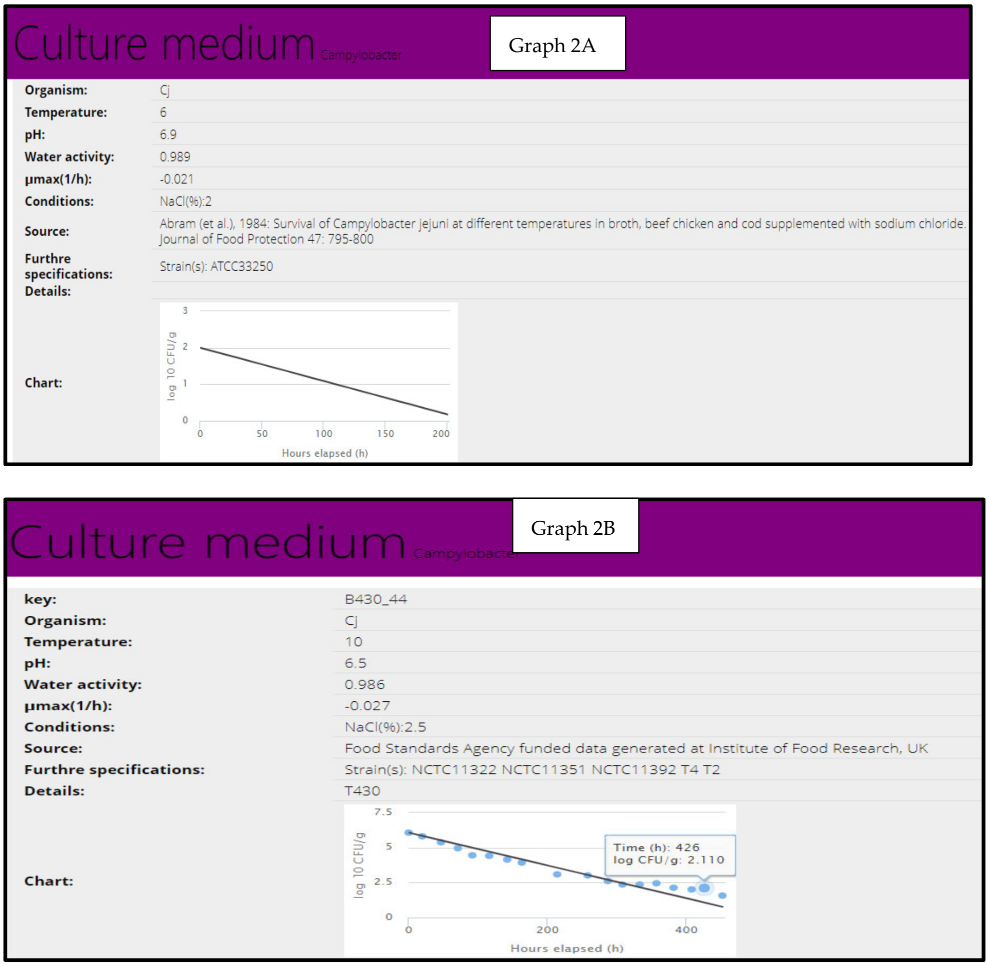

Figure S2. Details from Microbial Responses Viewer's time-dependent survival graphs (A and B) of *Campylobacter* spp.

Table S1. Wild and collection isolates of *Campylobacter jejuni* strains and their death kinetics, Weibull parameters and R-values in culture media.

Culture media									
**Strains**	**Temperature (˚C)**	**pH**	**a_w**	**μmax (1/h)**	**Conditions**	**First reduction time(h)**	**p value**	**R (correlation coefficient)**	**Data source**
NCTC11322; NCTC11351; NCTC11392; T4; T2	0	5,1	0,974	-0.041	NaCl(%):4.5	71,54632	1,217425	0,97491	*
T2; T4; NCTC11322; NCTC11351; NCTC11392	0	5,3	0,974	-0.019	NaCl(%):4.5, CO_2 (%):80	425,7484	1,258251	0,95536	*
T2; T4; NCTC11322; NCTC11351; NCTC11392	0	5,3	0,974	-0.007	NaCl(%):4.5, CO_2 (%):30	131,4062	0,855489	0,98299	*
NCTC11322; NCTC11351; NCTC11392; T4; T2	0	6,7	0,974	-0.039	NaCl(%):4.5	37,37575	0,755138	0,99464	*
NCTC11322; NCTC11351; NCTC11392; T4; T2	0	5,0	0,980	-0.035	NaCl(%):3.5	65,97206	1,100184	0,97191	*
NCTC11322; NCTC11351; NCTC11392; T4; T2	0	5,7	0,980	-0.022	NaCl(%):3.5	85,46472	0,862739	0,98269	*
T2; T4; NCTC11322; NCTC11351; NCTC11392	0	6,5	0,980	-0.005	NaCl(%):3.5, CO_2 (%):50	426,8274	0,928548	0,93822	*
NCTC11322; NCTC11351; NCTC11392; T4; T2	0	6,8	0,980	-0.035	NaCl(%):3.5	56,96894	0,960782	0,99119	*
T2; T4; NCTC11322; NCTC11351; NCTC11392	0	4,6	0,986	-0.042	NaCl(%):2.5	205,2864	1,020159	0,95915	*
NCTC11322; NCTC11351; NCTC11392; T4; T2	0	4,6	0,986	-0.012	NaCl(%):2.5, CO_2 (%):30	41,05679	0,877829	0,9635	*
NCTC11322; NCTC11351; NCTC11392; T4; T2	0	5,1	0,986	-0.029	NaCl(%):2.5	91,79473	1,154158	0,99124	*
NCTC11322; NCTC11351; NCTC11392; T4; T2	0	6,4	0,986	-0.034	NaCl(%):2.5	53,57232	0,833939	0,98031	*
NCTC11322; NCTC11351; NCTC11392; T4; T2	0	5,0	0,992	-0.022	NaCl(%):1.5	148,2826	1,400804	0,9661	*
NCTC11322; NCTC11351; NCTC11392; T4; T2	0	5,3	0,992	-0.017	NaCl(%):1.5	55,70136	0,638150	0,95105	*
NCTC11322; NCTC11351; NCTC11392; T4; T2	0	5,9	0,992	-0.016	NaCl(%):1.5	147,5667	0,913781	0,98559	*
T2; T4; NCTC11322; NCTC11351; NCTC11392	0	6,3	0,992	-0.009	NaCl(%):1.5, CO_2 (%):80	508,7021	1,488401	0,9914	*
T2; T4; NCTC11322; NCTC11351; NCTC11392	0	4,6	0,997	-0.190	NaCl(%):0.5, CO_2 (%):50	189,3338	0,827760	0,92309	*
NCTC11322; NCTC11351; NCTC11392; T4; T2	0	4,8	0,997	-0.072	NaCl(%):0.5	37,51941	1,138430	0,99479	*
NCTC11322; NCTC11351; NCTC11392; T4; T2	0	5,8	0,997	-0.009	NaCl(%):0.5	237,2269	0,985537	0,98843	*
T2; T4; NCTC11322; NCTC11351; NCTC11392	0	6,5	0,997	-0.019	NaCl(%):0.5, CO_2 (%):30	955,3778	8,303815	0,96784	*
NCTC11322; NCTC11351; NCTC11392; T4; T2	0	6,7	0,997	-0.012	NaCl(%):0.5	147,8719	0,755017	0,98606	*
Wild strains	4	6,9	0,961	-0.092	NaCl(%):6.5	4,465658	0,318575	0,99390	**
Wild strains	4	6,9	0,974	-0.051	NaCl(%):4.5	32,25649	0,487590	0,99789	**

NCTC11322; NCTC11351; NCTC11392; T4; T2	4	6,3	0,977	-0.005	NaCl(%):4, CO_2 (%):50	615,1236	1,316105	0,98181	*
NCTC11322; NCTC11351; NCTC11392; T4; T2	4	6,4	0,977	-0.012	NaCl(%):4, CO_2 (%):100	641,2220	2,083990	0,98403	*
NCTC11322; NCTC11351; NCTC11392; T4; T2	4	7,1	0,977	-0.015	NaCl(%):4	435,1144	1,799047	0,98357	*
Wild strains	4	6,9	0,986	-0.058	NaCl(%):2.5	69,76323	0,480490	0,98739	**
Wild strains	4	6,9	0,992	-0.032	NaCl(%):1.5	114,0922	0,61897	0,99853	**
Wild strains	4	6,9	0,995	-0.037	NaCl(%):1	0,026757	0,000032	0,61198	**
NCTC11322; NCTC11351; NCTC11392; T4; T2	4	6,8	0,997	-0.005	NaCl(%):0.5, CO_2 (%):50	1006,103	1,533874	0,98668	*
NCTC11322; NCTC11351; NCTC11392; T4; T2	8	6,4	0,977	-0.003	NaCl(%):4, CO_2 (%):50	951,1217	1,725765	0,97245	*
NCTC11322; NCTC11351; NCTC11392; T4; T2	8	6,5	0,977	-0.012	NaCl(%):4, CO_2 (%):100	511,1250	1,679616	0,99749	*
NCTC11322; NCTC11351; NCTC11392; T4; T2	8	6,9	0,977	-0.014	NaCl(%):4	226,5561	1,188962	0,99049	*
NCTC11322; NCTC11351; NCTC11392; T4; T2	8	7,4	0,997	-0.003	NaCl(%):0.5	848,0501	1,040819	0,96777	*
NCTC11322; NCTC11351; NCTC11392; T4; T2	10	4,7	0,961	-0.112	NaCl(%):6.5	12,36733	0,739603	0,95463	*
NCTC11322; NCTC11351; NCTC11392; T4; T2	10	5,3	0,961	-0.025	NaCl(%):6.5	117,908	1,397928	0,905	*
NCTC11322; NCTC11351; NCTC11392; T4; T2	10	5,4	0,961	-0.050	NaCl(%):6.5	41,42754	0,924331	0,96913	*
NCTC11322; NCTC11351; NCTC11392; T4; T2	10	5,6	0,961	-0.055	NaCl(%):6.5	31,15281	0,894470	0,93912	*
NCTC11322; NCTC11351; NCTC11392; T4; T2	10	6,0	0,961	-0.059	NaCl(%):6.5	29,85152	0,830301	0,93927	*
NCTC11322; NCTC11351; NCTC11392; T4; T2	10	7,0	0,961	-0.126	NaCl(%):6.5	5,460752	0,446300	0,90563	*
NCTC11322; NCTC11351; NCTC11392; T4; T2	10	7,0	0,961	-0.174	NaCl(%):6.5	1,833386	0,367246	0,89833	*
T2 T4 NCTC11322 NCTC11351 NCTC11392	10	4,3	0,974	-0.032	NaCl(%):4.5, CO_2 (%):50	126,0811	1,739799	0,98356	*
NCTC11322; NCTC11351; NCTC11392; T4; T2	10	4,6	0,974	-0.189	NaCl(%):4.5	5,440639	0,567754	0,98295	*
NCTC11322; NCTC11351; NCTC11392; T4; T2	10	5,1	0,974	-0.062	NaCl(%):4.5	16,99284	0,583944	0,9875	*
NCTC11322; NCTC11351; NCTC11392; T4; T2	10	5,5	0,974	-0.069	NaCl(%):4.5	26,88725	0,908141	0,98425	*
NCTC11322; NCTC11351; NCTC11392; T4; T2	10	5,9	0,974	-0.032	NaCl(%):4.5	95,54052	1,256810	0,97382	*
NCTC11322; NCTC11351; NCTC11392; T4; T2	10	6,2	0,974	-0.078	NaCl(%):4.5	48,92257	1,337659	0,99234	*
NCTC11322; NCTC11351; NCTC11392; T4; T2	10	6,7	0,974	-0.081	NaCl(%):4.5	40,71477	1,142119	0,98979	*
NCTC11351; NCTC11392; NCTC11322; T2; T4	10	4,0	0,980	-3.305	NaCl(%):3.5	0,049265	0,319820	0,833580	*
NCTC11351; NCTC11392; NCTC11322; T2; T4	10	4,0	0,980	-5.120	NaCl(%):3.5	0,215255	0,594601	0,946490	*

NCTC11351; NCTC11392; NCTC11322; T2; T4	10	4,4	0,980	-0.587	NaCl(%):3.5	4,538174	0,953505	0,988500	*
NCTC11351; NCTC11392; NCTC11322; T2; T4	10	4,5	0,980	-0.462	NaCl(%):3.5	1,712076	0,525139	0,978330	*
NCTC11322; NCTC11351; NCTC11392; T4; T2	10	4,6	0,980	-0.109	NaCl(%):3.5	8,552573	0,580363	0,962530	*
NCTC11351; NCTC11392; NCTC11322; T2; T4	10	5,0	0,980	-0.121	NaCl(%):3.5	48,17091	1,114921	0,97438	*
NCTC11322; NCTC11351; NCTC11392; T4; T2	12	6,4	0,977	-0.019	NaCl(%):4, CO_2 (%):50	572,7066	2,387077	0,99579	*
NCTC11322; NCTC11351; NCTC11392; T4; T2	12	6,6	0,977	-0.029	NaCl(%):4, CO_2 (%):100	434,4238	2,626735	0,99266	*
NCTC11322; NCTC11351; NCTC11392; T4; T2	12	6,9	0,977	-0.027	NaCl(%):4	97,53146	1,078530	0,97886	*
NCTC11322; NCTC11351; NCTC11392; T4; T2	12	5,9	0,997	-0.024	NaCl(%):0.5, CO_2 (%):50	0,002155	0,000002	0,124160	*
NCTC11322; NCTC11351; NCTC11392; T4; T2	12	7,3	0,997	-0.008	NaCl(%):0.5	734,1049	1,689350	0,98251	*
NCTC11351; NCTC11392; NCTC11322; T2; T4	15	5,0	0,961	-0.051	NaCl(%):6.5	47,37934	0,981989	0,95828	*
NCTC11351; NCTC11392; NCTC11322; T2; T4	15	5,3	0,961	-0.062	NaCl(%):6.5	12,09556	0,609656	0,96938	*
NCTC11351; NCTC11392; NCTC11322; T2; T4	15	5,4	0,961	-0.389	NaCl(%):6.5	0,367220	0,326560	0,919080	*
NCTC11351; NCTC11392; NCTC11322; T2; T4	15	6,3	0,961	-0.080	NaCl(%):6.5	25,86734	0,942553	0,97913	*
NCTC11351; NCTC11392; NCTC11322; T2; T4	15	7,0	0,961	-0.093	NaCl(%):6.5	15,52653	0,681184	0,96571	*
NCTC11322 NCTC11351 NCTC11392 T4 T2	15	6,4	0,974	-0.215	NaCl(%):4.5	16,78483	1,384345	0,98244	*
NCTC11351; NCTC11392; NCTC11322; T2; T4	15	6,2	0,980	-0.088	NaCl(%):3.5	5,967163	0,465512	0,934540	*
NCTC11351; NCTC11392; NCTC11322; T2; T4	15	6,3	0,980	-0.130	NaCl(%):3.5	7,097016	0,651927	0,965480	*
NCTC11322; NCTC11351; NCTC11392; T4; T2	15	6,3	0,980	-0.064	NaCl(%):3.5	18,69310	0,734463	0,99174	*
NCTC11322; NCTC11351; NCTC11392; T4; T2	15	6,7	0,980	-0.074	NaCl(%):3.5	16,89820	0,697591	0,99393	*
T2; T4; NCTC11322; NCTC11351; NCTC11392	15	6,8	0,980	-0.016	NaCl(%):3.5, CO_2 (%):30	168,2602	1,134826	0,98914	*
NCTC11351; NCTC11392; NCTC11322; T2; T4	15	7,0	0,980	-0.074	NaCl(%):3.5	13,71029	0,686514	0,93241	*
NCTC11351; NCTC11392; NCTC11322; T2; T4	15	7,0	0,980	-0.115	NaCl(%):3.5	25,08762	1,350498	0,97531	*
NCTC11322; NCTC11351; NCTC11392; T4; T2	15	6,0	0,986	-0.093	NaCl(%):2.5	15,17394	0,726786	0,95090	*
NCTC11322; NCTC11351; NCTC11392; T4; T2	15	6,1	0,992	-0.057	NaCl(%):1.5	87,02788	1,548284	0,98476	*
T2; T4; NCTC11322; NCTC11351; NCTC11392	15	6,2	0,992	-0.008	NaCl(%):1.5, CO_2 (%):30	393,4127	1,663558	0,98554	*
NCTC11322; NCTC11351; NCTC11392; T4; T2	15	6,4	0,992	-0.029	NaCl(%):1.5	95,28635	1,116364	0,99335	*

NCTC11322; NCTC11351; NCTC11392; T4; T2	15	6,7	0,992	-0.020	NaCl(%):1.5	130,6002	1,182506	0,96553	*
NCTC11322; NCTC11351; NCTC11392; T4; T2	15	5,6	0,997	-0.065	NaCl(%):0.5	91,58385	1,812849	0,98824	*
NCTC11322; NCTC11351; NCTC11392; T4; T2	15	6,0	0,997	-0.012	NaCl(%):0.5, CO_2 (%):50	171,5909	1,466249	0,9907	*
NCTC11351; NCTC11392; NCTC11322; T2; T4	15	6,8	0,997	-0.028	NaCl(%):0.5	67,31553	0,890654	0,96707	*
NCTC11351; NCTC11392; NCTC11322; T2; T4	20	5,3	0,961	-0.326	NaCl(%):6.5	6,383260	0,570522	0,909260	*
NCTC11351; NCTC11392; NCTC11322; T2; T4	20	5,5	0,961	-0.116	NaCl(%):6.5	11,00639	0,712601	0,93631	*
NCTC11351; NCTC11392; NCTC11322; T2; T4	20	6,1	0,961	-0.161	NaCl(%):6.5	4,166087	0,430884	0,869980	*
NCTC11322; NCTC11351; NCTC11392; T4; T2	20	5,2	0,974	-0.132	NaCl(%):4.5	7,511787	0,627960	0,994890	*
NCTC11322; NCTC11351; NCTC11392; T4; T2	20	5,4	0,974	-0.181	NaCl(%):4.5	3,990549	0,613952	0,966620	*
NCTC11322; NCTC11351; NCTC11392; T4; T2	20	6,7	0,974	-0.145	NaCl(%):4.5	8,590017	0,725491	0,996650	*
NCTC11322; NCTC11351; NCTC11392; T4; T2	20	6,5	0,977	-0.029	NaCl(%):4, CO_2 (%):50	167,1513	1,705036	0,99776	*
NCTC11322; NCTC11351; NCTC11392; T4; T2	20	5,7	0,980	-0.135	NaCl(%):3.5	13,98256	0,620136	0,97687	*
NCTC11322; NCTC11351; NCTC11392; T4; T2	20	6,3	0,980	-0.078	NaCl(%):3.5	8,480434	0,571281	0,97917	*
NCTC11351; NCTC11392; NCTC11322; T2; T4	20	6,9	0,980	-0.111	NaCl(%):3.5	20,32432	1,028696	0,97235	*
NCTC11322; NCTC11351; NCTC11392; T4; T2	20	5,9	0,986	-0.053	NaCl(%):2.5	39,69852	0,838743	0,97798	*
NCTC11322; NCTC11351; NCTC11392; T4; T2	20	6,4	0,986	-0.078	NaCl(%):2.5	24,73095	0,888000	0,98588	*
NCTC11322; NCTC11351; NCTC11392; T4; T2	20	5,9	0,992	-0.117	NaCl(%):1.5	24,73095	0,888000	0,95429	*
NCTC11322; NCTC11351; NCTC11392; T4; T2	20	6,5	0,992	-0.051	NaCl(%):1.5	27,30574	0,760212	0,94936	*
NCTC11322; NCTC11351; NCTC11392; T4; T2	20	7,0	0,992	-0.057	NaCl(%):1.5	33,61173	0,898724	0,95533	*
NCTC11322; NCTC11351; NCTC11392; T4; T2	20	5,9	0,997	-0.131	NaCl(%):0.5	105,0718	2,713980	0,94769	*
NCTC11322; NCTC11351; NCTC11392; T4; T2	20	6,1	0,997	-0.017	NaCl(%):0.5, CO_2 (%):50	245,7678	1,366024	0,99149	*
NCTC11322; NCTC11351; NCTC11392; T4; T2	20	6,5	0,997	-0.051	NaCl(%):0.5	56,54814	1,174313	0,95959	*
NCTC11322; NCTC11351; NCTC11392; T4; T2	20	6,7	0,997	-0.030	NaCl(%):0.5, CO_2 (%):100	176,6033	1,482621	0,99743	*
NCTC11351; NCTC11392; NCTC11322; T2; T4	20	6,8	0,997	-0.115	NaCl(%):0.5	10,55671	0,548010	0,93167	*
NCTC11322; NCTC11351; NCTC11392; T4; T2	25	5,5	0,974	-0.414	NaCl(%):4.5	3,221843	0,706566	0,99335	*
NCTC11322; NCTC11351; NCTC11392; T4; T2	25	6,4	0,974	-0.329	NaCl(%):4.5	7,106071	0,906223	0,97794	*

T2; T4; NCTC11322; NCTC11351; NCTC11392	25	6,8	0,974	-0.051	NaCl(%):4.5, CO_2 (%):50	33,70048	0,785043	0,98237	*
NCTC11322; NCTC11351; NCTC11392; T4; T2	25	5,3	0,980	-0.260	NaCl(%):3.5	10,12613	1,209200	0,99788	*
T2; T4; NCTC11322; NCTC11351; NCTC11392	25	6,0	0,980	-0.048	NaCl(%):3.5, CO_2 (%):30	42,49862	0,933558	0,99442	*
NCTC11322; NCTC11351; NCTC11392; T4; T2	25	6,6	0,980	-0.309	NaCl(%):3.5	5,838055	0,821535	0,98535	*
NCTC11322; NCTC11351; NCTC11392; T4; T2	25	5,9	0,986	-0.142	NaCl(%):2.5	6,495632	0,551287	0,98953	*
NCTC11322; NCTC11351; NCTC11392; T4; T2	25	6,6	0,986	-0.211	NaCl(%):2.5	7,039148	0,734165	0,97607	*
T2; T4; NCTC11322; NCTC11351; NCTC11392	25	5,9	0,992	-0.026	NaCl(%):1.5, CO_2 (%):50	140,7970	2,203209	0,98059	*
NCTC11322; NCTC11351; NCTC11392; T4; T2	25	6,3	0,992	-0.087	NaCl(%):1.5	30,31606	1,113888	0,99283	*
NCTC11322; NCTC11351; NCTC11392; T4; T2	25	4,5	0,997	-0.589	NaCl(%):0.5	3,755774	1,028277	0,99380	*
NCTC11322; NCTC11351; NCTC11392; T4; T2	25	5,5	0,997	-0.023	NaCl(%):0.5, CO_2 (%):30	49,55770	1,601693	0,98647	*
NCTC11322; NCTC11351; NCTC11392; T4; T2	25	5,9	0,997	-0.104	NaCl(%):0.5	44,37527	1,464024	0,98065	*

*Food Standards Agency funded data generated at Champden and Chorleywood Food Research Association, UK.

**Doyle (et al.), 1982: Response of *Campylobacter jejuni* to sodium chloride. Applied and Environmental Microbiology 43: 561-565.

Table S2. Wild and collection isolates of *Campylobacter jejuni* strains and their death kinetics, Weibull parameters and R-values in poultry.

**Poultry**									
**Strains**	**Tempera-ture(°C)**	**pH**	**a_w**	**μmax (1/h)**	**Conditions**	**First reduc-tion time(h)**	***p* value**	**R (correla-tion coeffi-cient)**	**Data source**
H-840	4	6	-	-0.012	NaCl(%):0.5, cut	278,9079	1,542452	0,96776	***
ATCC 29428; CJ-B4086	4	6,5	0,995	-0.034	NaCl(%):0.85, CO_2(%):60	84,34400	1,162034	0,98844	****
ATCC 29428; CJ-B4087	4	6,5	0,995	-0.048	NaCl(%):0.85, N_2(%):100	61,43394	0,982495	0,98867	****
ATCC 29428; CJ-B4088	4	6,5	0,995	-0.052	NaCl(%):0.85, CO_2(%):40	60,23565	0,942862	0,97092	****
ATCC 29428; CJ-B4089	4	6,5	0,995	-0.036	NaCl(%):0.85, CO_2(%):100	100,7489	1,760718	0,99909	****
ATCC 29428; CJ-B4090	4	6,5	0,995	-0.036	NaCl(%):0.85, CO_2(%):80	124,4701	2,133446	0,99992	****
Wild strains	5	6,1	-	-0.020	irradiated	401,6508	1,354264	0,98267	*****
Wild strains	5	6,1	-	-0.027	ascor-bic_acid(ppm):650, irradiated	182,3127	1,000641	0,97487	*****
Wild strains	5	6,1	-	-0.029	irradiated	146,1828	0,886418	0,97465	*****
ATCC 29428; CJ-B4086	21	6,5	0,995	-0.242	NaCl(%):0.85	4,088950	0,680034	0,984370	****
ATCC 29428; CJ-B4087	21	6,5	0,995	-0.129	NaCl(%):0.85	19,10096	1,728139	0,99648	****
ATCC 29428; CJ-B4088	21	6,5	0,995	-0.243	NaCl(%):0.85	10,79891	0,702156	0,92196	****

ATCC 29428; CJ-B4089	21	6,5	0,995	-0.170	NaCl(%):0.85	21,91347	1,104969	0,94201	****
ATCC 29428; CJ-B4090	21	6,5	0,995	-0.115	NaCl(%):0.85	10,17826	0,829917	0,97796	****
H-840	23	6	-	-0.036	NaCl(%):0.5, cut	53,09475	1,168183	0,97923	***
B8852	23	6	-	-0.019	NaCl(%):0.5, cut	67,13805	0,743855	0,9742	***
B8788	23	6	-	-0.013	NaCl(%):0.5, cut	198,5271	1,158213	0,98423	***

The wild strains of *C. jejuni* included the following: FRI-CF3, FRI-CF6, and FRI-CF8, which were of human origin; FRI-CF33P, which was of porcine origin; FRI-CF74C, which was of avian origin; and FRICF145B and FRI-CF147B, which was of bovine origin. The NARTC strain, FRI-CF31P, was of porcine origin. ***Blankenship (et al.), 1982: *Campylobacter jejuni* survival in chicken meat as a function of temperature. Applied and Environmental Microbiology 44: 88-92. ****Phebus (et al.), 1991: Survival of *Campylobacter jejuni* in modified atmosphere packaged turkey roll. Journal of Food Protection 54: 194-199. *****Juven (et al.), 1988: Effect of ascorbic and isoascorbic acids on survival of Campylobacter jejuni in poultry meat. Journal of Food Protection 51: 436-437.
